# Parasitoid Increases Survival of Its Pupae by Inducing Hosts to Fight Predators

**DOI:** 10.1371/journal.pone.0002276

**Published:** 2008-06-04

**Authors:** Amir H. Grosman, Arne Janssen, Elaine F. de Brito, Eduardo G. Cordeiro, Felipe Colares, Juliana Oliveira Fonseca, Eraldo R. Lima, Angelo Pallini, Maurice W. Sabelis

**Affiliations:** 1 Institute for Biodiversity and Ecosystem Dynamics, Section Population Biology, University of Amsterdam, Amsterdam, The Netherlands; 2 Department of Animal Biology, Section Agricultural Entomology, Federal University of Viçosa, Minas Gerais, Brazil; Queen Mary College, University of London, United Kingdom

## Abstract

Many true parasites and parasitoids modify the behaviour of their host, and these changes are thought to be to the benefit of the parasites. However, field tests of this hypothesis are scarce, and it is often unclear whether the host or the parasite profits from the behavioural changes, or even if parasitism is a cause or consequence of the behaviour. We show that braconid parasitoids (*Glyptapanteles sp.*) induce their caterpillar host (*Thyrinteina leucocerae*) to behave as a bodyguard of the parasitoid pupae. After parasitoid larvae exit from the host to pupate, the host stops feeding, remains close to the pupae, knocks off predators with violent head-swings, and dies before reaching adulthood. Unparasitized caterpillars do not show these behaviours. In the field, the presence of bodyguard hosts resulted in a two-fold reduction in mortality of parasitoid pupae. Hence, the behaviour appears to be parasitoid-induced and confers benefits exclusively to the parasitoid.

## Introduction

Diseases, parasites and parasitoids can induce spectacular changes in the behaviour of their host [1]–[11]. Some of these changes, such as behavioural fevering [12] and exposure to cold temperatures [13], are thought to benefit the host, but others have been suggested to result in increased transmission of parasites [1], [3], [4], [14]–[17] or increased survival of parasitoids [18]–[22]. One of the most famous examples is the parasitic trematode *Dicrocoelium dendriticum*, which induces its intermediate host, ants, to move up onto blades of grass during the night and early morning, and firmly attach themselves to the substrate with their mandibles [3]. This is believed to enhance parasite transmission due to increased ingestion of infected ants by grazing sheep, the final host [23]. In contrast, uninfected ants return to their nests during the night and the cooler parts of the day. Other examples of such spectacular behavioural changes include parasitoid larvae (*Hymenoepimecis* sp.) that induce their spider host (*Plesiometa argyra*) to construct a special cocoon web in which the larvae pupate [7], rodents infected by *Toxoplasma* that lose their innate aversion to odours of cats, the parasite's final host [9], and hairworms that induce their terrestrial arthropod hosts to commit suicide by jumping into water, after which the hairworms desert the host to spend their adult stage in their natural habitat [6], [8].

Although many of these examples are consistent with host manipulation, concern has been voiced over this interpretation of the existing evidence [1], [2], [4]. For example, supporting evidence for increased transmission of parasites comes mainly from laboratory studies and consists of correlations between behavioural changes and a higher risk of predation of intermediate hosts by the final host [4], [5]. Obviously, fitness consequences for the host and parasite should be evaluated under field conditions, where the host-parasite complex may also suffer increased predation from organisms that are not hosts of the parasite [1], [4], [14], [24].

The key problem with field experiments is the difficulty in assessing whether a behavioural change is adaptive for the parasite, adaptive for the host, or actually represents a non-adaptive and/or accidental pathological side-effect resulting from infection of the host [1], [4], [10], [18], [25]. Moreover, it is possible that parasites more readily infect or parasitize hosts that behave differently to conspecifics [4], [25]. In the latter case, the observed behaviour would not be a consequence, but rather a cause, of parasitism.

In contrast to the case of true parasites [1], [2], [4], behavioural changes in parasitoid hosts are hypothesized to result in increased parasitoid survival through decreased host predation [19]–[22], [26], because parasitoids typically die with the host. Although such behavioural manipulation of hosts by parasitoids has been reported frequently [19]–[22], [27], [28], field evidence for the advantages of the behavioural change for parasitoids is even scarcer than for true parasites [10], [18], [21], and is also constrained by the possibility that parasitoids selected hosts with aberrant behaviour [4].

**Figure 1:**
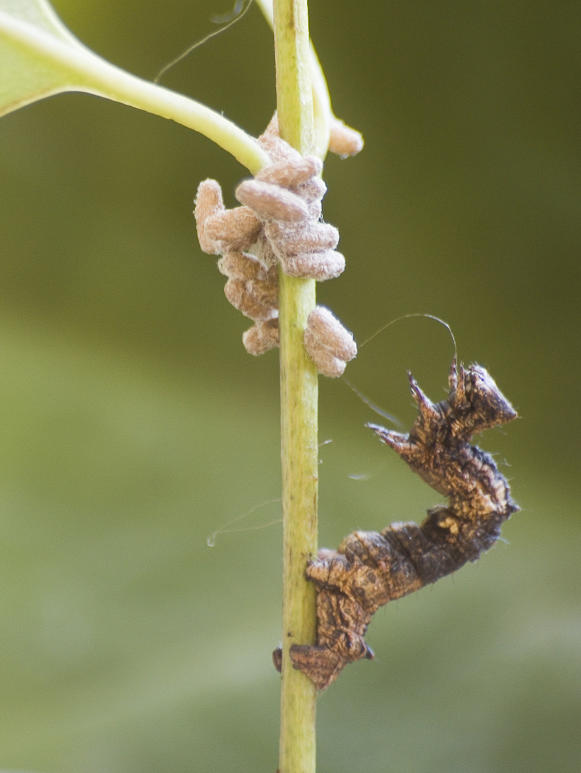
A caterpillar of the geometrid moth *Thyrinteina leucocerae* with pupae of the Braconid parasitoid wasp *Glyptapanteles* sp. Full-grown larvae of the parasitoid egress from the caterpillar and spin cocoons close by their host. The host remains alive, stops feeding and moving, spins silk over the pupae, and responds to disturbance with violent head-swings (supporting information). The caterpillar dies soon after the adult parasitoids emerge from the pupae. Photograph by Prof. José Lino-Neto.

In this study we present evidence for behavioural changes in a host that are beneficial to its parasitoid under field conditions. We studied the consequences of behavioural manipulation of the geometrid moth *Thyrinteina leucocerae* by its parasitoid wasp (*Glyptapanteles* sp., Braconidae) on parasitoid survival in the field in Brazil. Adult female parasitoids oviposit in first- and second-instar caterpillars of the moth, which feed on foliage of various trees of the Myrtaceae family, such as guava and eucalyptus. Parasitized caterpillars continue developing and feeding until the 4^th^ or 5^th^ instar, when up to c. 80 full-grown parasitoid larvae egress from the host to pupate (A.H. Grosman and A. Janssen, pers. obs.). The larvae spin cocoons on a twig or leaf close to the caterpillar and pupate (Fig. 1). Subsequently, the host undergoes a series of behavioural changes, including cessation of feeding and moving. The most profound change in behaviour, however, is a strong increase of violent head-swings upon disturbance, in an apparent attempt to hit the agent of disturbance (A.H. Grosman and A. Janssen, pers. obs.). It has been suggested that such head-swings could serve as a defence of the parasitoid pupae against predation or hyperparasitism [20], [22], but evidence is lacking. We therefore quantified the effects of these behavioural changes on interactions with predators in the laboratory, as well as on survival of the parasitoid pupae in the field.

## Materials and Methods

*Thyrinteina leucocerae* and *Glyptapanteles* sp. were collected from guava (*Psidium guajava*) and *Eucalyptus grandis* trees on the campus of the Federal University of Viçosa, Minas Gerais, Brazil (20°45′ S, 42°51′ W). The parasitoid species awaits further taxonomic description, and voucher specimens are deposited with Prof. A. Menezes Jr. at the University of Londrina, Brazil. Caterpillars were reared either in groups on small eucalyptus or guava trees (30–90 cm high) in cages (70×70 cm, 100 cm high) outside the laboratory, or individually in plastic cups (500 ml) in the laboratory at ambient temperature and light conditions. The cups contained small (5–10 cm) twigs of eucalyptus or guava with some 1–7 leaves, and were closed with a mesh. The twigs were inserted into moist vermiculite to maintain leaf turgor. Fresh twigs were added twice per week. Moth pupae were transferred to cages (as above) outside the laboratory, each containing a small tree and filter paper moistened with a solution of honey in water (10% v/v). Moths were allowed to emerge and adults mated and oviposited inside the cages. Eggs were collected from the cages once a week, and were left to emerge in cages containing small trees. The host cultures were frequently supplemented with field-collected individuals.

Recently emerged adult parasitoids, one female and 1–2 males, were incubated for 24 hours in a glass tube containing a piece of host plant leaf to allow them to mate. They were subsequently placed in glass tubes (containing agar and some honey, closed with foam rubber) and either kept in the laboratory when caterpillars were available or stored in a climate box (12°C±3, L12:D12) until there was a supply of caterpillars. Subsequently, the adult parasitoids were incubated for 24 hours in a plastic cup (500 ml) containing some leaves and up to 8 first-instar *T. leucocerae* caterpillars of the same age. Parasitism is very rapid, occurring as a female parasitoid apparently walks over a host caterpillar. Immediate dissection of the caterpillar reveals up to 80 eggs inside (A. Janssen, pers. obs.). Parasitoid larvae egress from parasitized caterpillars through exit holes they make in the host cuticle and pupate after 11–16 days (A.H. Grosman, pers. obs). Parasitoid pupae were collected from the cups and incubated in glass tubes in the laboratory until adult emergence. As with the host, the parasitoid cultures were frequently supplemented with field-collected individuals.

For all experiments, we used caterpillars emerging from the same egg batches, which were subdivided into groups: one group was exposed to parasitoids to obtain parasitized caterpillars, whereas the other group was not exposed (i.e. caterpillars remained unparasitized). Because each group had an equal probability of containing hosts with aberrant behaviour, this minimized the possibility that any behavioural changes observed were due to parasitoids selecting hosts with atypical behaviour, rather than a consequence of parasitism [4].

### Effect of parasitism on host locomotion

First-instar hosts (parasitized and unparasitized) were placed individually on small *E. grandis* trees (c. 50 cm high) in cages outside the laboratory. Caterpillars were prevented from walking off the plant using a ring of insect glue (Cola Entomológica, Bio Controle, São Paulo, Brazil) applied to the stem of the seedlings. Replicates in which the caterpillar disappeared (<16%) were discarded. Upon egression, half of the twigs with parasitoid pupae were cut off, while the caterpillar was left undisturbed on the plant. The twigs with pupae were stapled to a leaf close by an unparasitized caterpillar, resulting in four treatments: parasitized and unparasitized caterpillars either with or without parasitoid pupae close by. We marked the position of the caterpillars by tying a thin thread on the plant just behind the abdominal prolegs, taking care not to disturb the caterpillars. Each subsequent day, we measured the distance moved by the caterpillar from the original thread (by tying another thread just behind the abdominal prolegs). Caterpillar locomotion was scored until either five days after parasitoid egression or five days after the addition of parasitoid pupae. Caterpillar size was measured in a similar way with another piece of thread. Locomotion of unparasitized caterpillars without pupae was scored until 5 days after the average caterpillar age at parasitoid egression (23 days). Although no parasitoid larvae egressed from unparasitized caterpillars, for brevity we refer to the movement of parasitized and unparasitized host before and after egression in all treatments. The distribution of movement data was non-normal due to zero inflation, even after transformations; we therefore used the more conservative non-parametric Kruskal-Wallis test [29] to compare locomotion among treatments before or after parasitoid egression. A Wilcoxon matched pairs test [29] was used to compare caterpillar locomotion before and after egression within treatments [29] using R statistical software (R, version 2.3.1, 2006. R Development Core Team 2006, R Foundation for Statistical Computing, Vienna, Austria). Caterpillar body length was compared using a t-test.

### Defensive behaviour in the laboratory

We used third-instar stinkbugs (*Supputius cincticeps* (Stäl), Heteroptera, Pentatomidae) to quantify the response of parasitized and unparasitized hosts to predators. Predators of this genus attack parasitoid pupae as well as *T. leucocerae* caterpillars in the field (A.H. Grosman, pers. obs.). Predators were obtained from a mass culture at the Federal University of Viçosa fed with *Tenebrio molitor* L. larvae and were individually incubated for one day in Petri dishes (14 cm diameter) containing a source of water (a moist piece of cotton wool) and some parasitoid pupae to familiarize predators with pupae as food. Subsequently, they were incubated for another day without parasitoid pupae to starve them, thus increasing their tendency to search for prey.

Twigs with unparasitized or parasitized caterpillars with their pupae were inserted into a foam block, so that the twig was positioned vertically. A starved predator was introduced gently at some 2–4 cm from the caterpillar without disturbing the latter, and was allowed to search. It was reintroduced if it left the twig before encountering the caterpillar or pupae. Parasitized and unparasitized caterpillars were tested in an alternate sequence, and each caterpillar and each predator was tested once. Average observation time was 5.4±0.87 min (mean±s.e.m.) for parasitized caterpillars and 6.7±0.87 min for unparasitized caterpillars. When the predator encountered the caterpillar, we scored the number of head-swings the caterpillar directed towards the predator, as well as the outcome of the interaction (escape of the predator, predator knocked off by the head-swings). The number of head-swings by parasitized and unparasitized caterpillars were compared with a generalized linear model with quasi-Poisson error distribution to correct for overdispersion [30], using R statistical software. The numbers of predators that gave up or were chased away by the defending caterpillar were compared with a Fisher's exact test [29].

### Effect of host on parasitoid pupa mortality in the field

Field experiments were carried out from 1 July to 17 August 2005 in two guava plantations on the campus of the Federal University of Viçosa. The vegetation covering the soil consisted mainly of grasses; the plantations were surrounded by more diverse native vegetation. One of the guava plantations was managed organically; the other plantation was not managed.

We obtained parasitized caterpillars as described above. All batches of parasitoid pupae that emerged on the same day were placed in the same field within one day of egression and pupation of the parasitoids. The guarding caterpillar was removed from 43% of the batches. Each batch was attached to a separate guava tree by stapling the twig (with or without caterpillar, depending on the treatment) to a leaf, thus exposing it to predators and parasitoids. The number of pupae in batches with and without host did not differ significantly between treatments (with host: 35.5±1.8, without host: 33.1±2.0, t-test, P = 0.37). A total of 118 batches of parasitoid pupae were exposed in the two guava plantations.

To measure mortality due to causes other than predation and hyperparasitism, we covered branches, to which twigs with pupae and caterpillars were attached, with a sleeve cage of fine mesh (below referred to as unexposed batches). Insect glue applied to the base of each branch prevented walking predators and parasitoids from accessing these unexposed batches. Batches were recollected after three days (c. half of the pupal period), pupae were counted, and the presence or absence of the caterpillar recorded. Pupae were subsequently incubated for one month (25°C±5, L12:D12) to allow emergence of parasitoids and hyperparasitoids. The proportion of pupae per batch which were eaten by predators or hyperparasitized was compared among treatments using GLM with quasi-binomial error distributions to correct for overdispersion [30], using R statistical software.

## Results

### Effect of parasitism on host locomotion

**Figure 2:**
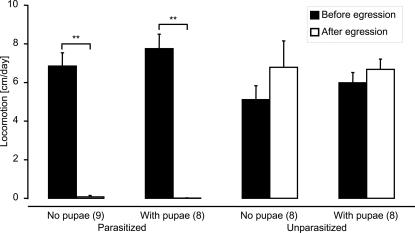
Effect of parasitism on host locomotion on the plant. The distance covered by parasitized and unparasitized caterpillars was measured daily. Parasitoid pupae were either removed from parasitized caterpillars (No pupae) or not (With pupae). Unparasitized caterpillars were supplied with pupae (With pupae) or not (No pupae). Before parasitoid egression (black bars: mean+s.e.m.), the difference in displacement of parasitized and unparasitized caterpillars was not significant (Kruskal-Wallis test, KW = 7.12, d.f. = 3, P = 0.068). After egression (white bars: mean+s.e.m.), parasitized caterpillars moved significantly less far than unparasitized caterpillars (KW = 24.0, d.f. = 3, P<0.001). The difference in displacement of parasitized caterpillars before and after egression was significant (**: Wilcoxon Matched Pairs test, P<0.01). Numbers of replicates are given in brackets.

Before egression of the parasitoid larvae, parasitized and unparasitized caterpillars did not differ in body length (parasitized: n = 17, 2.84±014 cm (mean±s.e.m.); unparasitized: n = 17, 3.00±0.08 cm, t-test: t = 0.995, P = 0.33). All caterpillars moved, and although parasitized caterpillars moved more than unparasitized caterpillars (7.3±0.50 and 5.6±0.45 cm/day respectively), there were no significant differences in movement among treatments (Fig. 2, Kruskal Wallis test: KW = 7.12, d.f. = 3, P = 0.068).

Fifteen out of 17 (88%) parasitized caterpillars stopped feeding and moving over the plant within one day after the parasitoids had egressed (and pupated), and all remained close to the parasitoid pupae, standing on their two pairs of abdominal prolegs, often bent over the cluster of pupae (Fig. 1). The two parasitized caterpillars that moved following parasitoid egression (one with pupae and one without pupae) covered a distance of 0.12 and 0.67 cm respectively. There was a highly significant difference in distances travelled by caterpillars before and after parasitoid egression (Wilcoxon Matched Pairs test: V = 153, P<0.001). All parasitized caterpillars died soon after the adult parasitoids emerged from the pupae, some 6–7 days after egression of the larvae. This shows that the behavioural changes described here (and below) do not benefit the parasitized host.

In contrast, all unparasitized caterpillars continued feeding and moving, any difference in locomotion before and after the time at which egression would have taken place (had they been parasitized) was not significant (Wilcoxon Matched Pairs test: V = 44, P = 0.23). The difference in the number of parasitized and unparasitized caterpillars moving after the time of parasitoid egression was highly significant (Fisher's exact test: p<0.0001), as was the difference in distance travelled (Fig. 2, KW = 24.0, d.f. = 3, P<0.001).

There were no significant differences in distance travelled comparing either parasitized (Kruskal Wallis test: KW = 0, d.f. = 1, Bonferroni-corrected P = 1) or unparasitized (KW = 0.011, d.f. = 1, Bonferroni-corrected P = 0.92) caterpillars with and without pupae. This indicates that the presence of parasitoid pupae does not induce a change in host behaviour.

### Defensive behaviour in the laboratory

**Figure 3:**
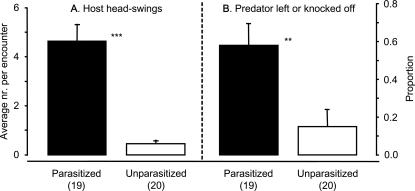
Effect of parasitism on host-predator interactions in the laboratory. A predator was introduced on a twig, 2–4 cm away from a parasitized or unparasitized caterpillar, without disturbing the caterpillar. A. Upon being encountered by a predator, parasitized caterpillars (black bars: mean+s.e.m.) swung their heads more frequently than unparasitized (white bars: mean+s.e.m.) caterpillars (***: GLM with quasi-Poisson errors, F_1,37_ = 57.6, P<0.001). B. The proportion of predators that gave up or were knocked off the twig was higher for parasitized compared with unparasitized hosts (**: Fisher's Exact Test, P = 0.008). Numbers of replicates are given in brackets.

When detecting a predator that was introduced on the twig, 17 out of 19 parasitized caterpillars lashed out at the bug with repeated violent head-swings (see Movie S1). Only one of 20 unparasitized caterpillars showed this behaviour, whereas the others hardly responded to the presence of the predator, even when it was walking on the host (see Movie S2). The difference in the number of parasitized and unparasitized caterpillars that showed head-swings was highly significant (Fisher's exact test: P<0.0001). Prior to parasitoid egression, parasitized caterpillars also do not respond to disturbance with head-swings (A.H. Grosman and A. Janssen, pers. obs.). Parasitized caterpillars showed a significantly higher number of head-swings towards the predator than unparasitized caterpillars (Fig. 3A, GLM with quasi-Poisson errors, F_1,37_ = 57.6, P<0.001). In more than half of the encounters of a predator with a parasitized caterpillar, the repeated head-swings caused the predators either to give up and leave the twig or to be knocked off (Fig. 3B), and the predators succeeded in contacting the pupae in only 35% of the interactions. Predators were never knocked off by unparasitized caterpillars, and gave up in only 15% of the cases (Fig. 3B, difference between parasitized and unparasitized caterpillars: Fisher's exact test, P = 0.008).

### Effect of host on parasitoid pupa mortality in the field

In the field, parasitoid pupae were readily attacked by various ant species, predatory bugs such as *Supputius spp.*, and four species of hyperparasitoid wasps. Significantly more pupae were damaged or disappeared from batches of pupae that were exposed to predators and parasitoids than from unexposed batches in sleeve cages (average mortality per batch: unexposed = 4.2%±1, exposed: 26.6%±3.2, GLM, F_1,132_ = 10.5, P<0.005). We scored predation in the exposed batches as the proportion of pupae per batch that had disappeared or was damaged.

**Figure 4:**
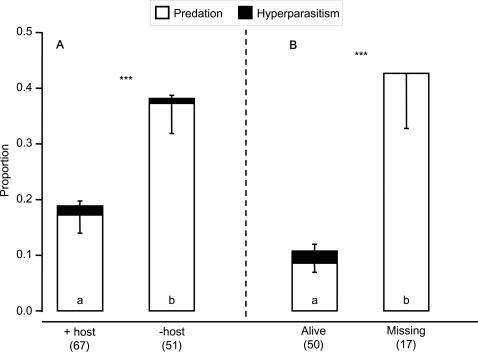
Effect of removing the guarding host on field mortality of parasitoid pupae. Twigs with known numbers of parasitoid pupae were attached to a leaf of a guava tree (each batch to a different tree) mimicking the natural situation. The guarding caterpillar was removed at random from 43% of the batches of parasitoid pupae. A. Total mortality, expressed as mean proportion of pupae per batch eaten by predators (white bars: mean−s.e.m.) or hyperparasitized (black bars: mean+s.e.m.). The mean proportion of pupae lost per batch (presumably eaten by predators) was significantly lower in the presence of the host (+ host) than when the caterpillar was absent (- host) (total: ***: GLM with quasi-binomial errors, F_1,116_ = 8.25, P<0.005, predation: F_1,116_ = 8.85, P<0.005). Levels of hyperparasitism per batch were not significantly different in the presence or absence of the host (F_1,116_ = 0.09, P = 0.76). B. Of the batches of pupae with host (+ host in A), total mortality and predation with a live host was lower than when the host was missing at the end of the period of field exposure (total: **: F_1,65_ = 23.9, P<0.0001, predation: F_1,65_ = 32.7, P<0.0001), but hyperparasitism did not differ significantly between treatments (F_1,65_ = 2.78, P = 0.10). Numbers of replicates are given in brackets.

Removal of the caterpillars resulted in a two-fold increase in mortality of batches of parasitoid pupae (Fig. 4A, GLM, F_1,116_ = 8.25, P<0.005). Contrary to what has been suggested [19], this was mainly due to differences in predation (Fig. 4A, F_1,116_ = 8.85, P<0.005) and not hyperparasitism, which accounted for only 3.1 (±0.8) % mortality and did not differ between treatments (Fig. 4A. F_1,116_ = 0.09, P = 0.76). Caterpillars disappeared from 25% of the (exposed) batches of parasitoid pupae in the field. This is likely to be due to predation because parasitized caterpillars hardly move once parasitoid larvae egress (Fig. 2), and caterpillars inside sleeve cages did not disappear. The mortality in batches of parasitoid pupae from which the caterpillars disappeared was as high as that in batches from which caterpillars were experimentally removed (Fig. 4, F_1,66_ = 0.27, P = 0.60), and much higher than in batches from which the caterpillar survived the period of field exposure (Fig. 4, F_1,65_ = 23.9, P<0.0001). We do not know whether death of these pupae occurred before or after the disappearance of the caterpillar, or was actually causally related to it. Possibly, some predators were attracted by the caterpillar and subsequently also fed on the parasitoid pupae. If this were the case, this suggests that there may also be costs involved with the behavioural changes in the caterpillar: behavioural changes might attract some predators against which the caterpillar cannot defend the parasitoid pupae. Nevertheless, the overall effect of caterpillar presence on survival of parasitoid pupae was positive (Fig. 4A).

## Discussion

The behaviour of parasitized hosts changed dramatically after the egression and pupation of parasitoid larvae. Hosts stopped walking and feeding and remained near parasitoid pupae. In addition, they performed 10 times more head-swings than unparasitized hosts during encounters with predators. As a result, predators were deterred in 58% of the encounters with parasitized hosts, but gave up in only 15% of the encounters with unparasitized hosts. It could be argued that this behavioural change serves the parasitoids as well as the host, because both would suffer less predation. However, the guarding caterpillar always died shortly after the adult parasitoids emerged from their pupae. Thus increased caterpillar survival during the period in which parasitoids pupate does not result in increased host fitness. Hence, the hosts appear to behave as a bodyguard of the parasitoid pupae.

The field experiment further confirmed that parasitoid pupae indeed suffered less predation in presence of their host. Host defence of parasitoid pupae was ineffective against hyperparasitoids, but this did not appear to represent an important parasitoid mortality factor. Possibly, these specialized natural enemies have adapted to the defending host. We conclude that the parasitoids, and not the hosts, benefited from the behavioural changes of the host that appear to be induced by the parasitoids.

It is unlikely that parasitoids select hosts that showed atypical behaviour at the time of parasitism as we used unparasitized and parasitized caterpillars emerging from the same batches of eggs. The sudden cessation of movement and feeding of parasitized caterpillars upon parasitoid egression, the increased number of head-swings, and the total lack of such behavioural changes in unparasitized caterpillars further confirms this. Hence, the behavioural changes described here are consistent with the hypothesis that they are induced by the parasites. This begs for an explanation of how the parasitoid induces behaviour changes in its host and which stage induces it. Given the long time (2 weeks) between parasitism and the behavioural change, the adult parasitoid is not likely to be the inducer. Furthermore, the changes in host locomotion behaviour were not induced by stimuli from the parasitoid pupae, because removal of the pupae from parasitized hosts or adding pupae to unparasitized host did not alter or induce the behavioural changes. Moreover, the mechanical damage caused by egressing parasitoid larvae is probably not the cause of the behavioural change. In pilot experiments, artificially damaging unparasitized hosts did not induce modified behaviour (F. Colares pers. obs.).

Parasitoid larvae are known to interfere with host endocrine functions, causing the host to stop feeding before parasitoid larvae egress [10], [28], [31]–[35]. Levels of juvenile hormone, ecdysteroids and neurotransmitters (e.g. octopamine) have been found to increase shortly before parasitoid egression [33]–[35]. However, it is not clear whether parasitoid larvae produce these substances in sufficient quantity to change host behaviour [10], [34]. Moreover, the most important behavioural changes in the present study occur only after the parasitoids have egressed. The egression usually takes about 1 hour, and the caterpillars do not respond strongly to disturbance during egression, but only 1–2 hours after the event. This casts doubt on the role of the parasitoid larvae in the behavioural changes. However, when we dissected caterpillars from which parasitoids had egressed 3–4 days before, we found 1–2 active parasitoid larvae that had remained behind in the host, as has been found in another system [36]. We hypothesise that these parasitoid larvae are responsible for the changes in host behaviour. A similar mechanism has been described for the trematode *D. dendriticum* [37] and the liver fluke *Brachylecithum mosquensis* [23], which both use ants as an intermediate host. One or two of the parasites migrate to the ant's brain, where they encyst and are believed to affect the ant's behaviour. These so-called brainworms are not transmitted, and appear to be sacrificed to enable transmission of their kin [38]. If the parasitoid larvae of the system described here also stay behind to manipulate the host and do not pupate later, this would represent a cost of host manipulation: some offspring are sacrificed for higher survival of their kin [39]. This hypothesis needs further investigation.

There has been considerable debate on behavioural changes of hosts being true manipulations by the parasitoid or by-products of infection [2], [4]. Although we do not yet know the mechanisms that induce behavioural changes in our system, it is clear that the modified behaviour is beneficial to the parasitoid. Hence, even if behavioural changes were initially by-products of infection, parasitoids would be strongly selected to induce these by-products more effectively, and it would be currently impossible to distinguish between ‘coincidentally beneficial’ by-products and parasitoid adaptation [1].

## Supporting Information

Movie S1 A parasitized caterpillar, bent over the parasitoid pupae that have egressed from it, defends itself and the parasitoid pupae against a predator with violent head-swings, resulting in the predator being knocked off the twig.
(2.59 MB WMV)

Movie S2 A non-parasitized caterpillar hardly responds to a predator
(1.43 MB WMV)
